# Successfully resisting a pathogen is rarely costly in *Daphnia magna*

**DOI:** 10.1186/1471-2148-10-355

**Published:** 2010-11-17

**Authors:** Pierrick Labbé, Pedro F Vale, Tom J Little

**Affiliations:** 1 University of Edinburgh, Institute of Evolutionary Biology, King's Buildings, Edinburgh, EH9 3JT, UK; 2 University of Montpellier 2, Institut des Sciences de l'Evolution de Montpellier, Pl Eugène Bataillon, CC065, 34095 Montpellier cedex 05, France; 3 Centre d'Ecologie Fonctionnelle et Evolutive (CEFE) - UMR 5175 1919 route de Mende, 34293 Montpellier, France

## Abstract

**Background:**

A central hypothesis in the evolutionary ecology of parasitism is that trade-offs exist between resistance to parasites and other fitness components such as fecundity, growth, survival, and predator avoidance, or resistance to other parasites. These trade-offs are called costs of resistance. These costs fall into two broad categories: constitutive costs of resistance, which arise from a negative genetic covariance between immunity and other fitness-related traits, and inducible costs of resistance, which are the physiological costs incurred by hosts when mounting an immune response. We sought to study inducible costs in depth using the crustacean *Daphnia magna *and its bacterial parasite *Pasteuria ramosa*.

**Results:**

We designed specific experiments to study the costs induced by exposure to this parasite, and we re-analysed previously published data in an effort to determine the generality of such costs. However, despite the variety of genetic backgrounds of both hosts and parasites, and the different exposure protocols and environmental conditions used in these experiment, this work showed that costs of exposure can only rarely be detected in the *D. magna-P. ramosa *system.

**Conclusions:**

We discuss possible reasons for this lack of detectable costs, including scenarios where costs of resistance to parasites might not play a major role in the co-evolution of hosts and parasites.

## Background

Parasites are thought to be a major cause of evolutionary change due to the deleterious fitness effects they impose on their hosts [1-3]. Coevolution between hosts and parasites has resulted in the evolution of several mechanisms to avoid or limit these deleterious effects, including behavioural modifications, boundary defences (e.g. the cuticle) and finally the immune system [reviewed in [4]]. Following theory on the evolution of life-history traits [5], the evolution of the immune system is thought to be shaped by costs of resistance, as investment in fighting infection by mounting and then maintaining an immune response should divert resources from other fitness-related traits [4,6-10].

These costs fall into two broad categories. Constitutive costs of resistance arise from a negative genetic covariance between immunity and other fitness-related traits (a genetic-based trade-off) [4,7,9,10]. Inducible costs of resistance are the physiological costs incurred by hosts when mounting an immune response [4,9,10]. Such inducible costs of mounting an immune response can be measured by comparing the fitness of individuals that are challenged with infection but successfully fight it off, to the fitness of hosts that are unchallenged [7]. The mechanistic cause of these costs (induced or constitutive) is thought to be the energy requirements necessary to fight infection, but they could also be linked to direct deleterious effects of immune effectors on the host itself (that is, immunopathology [see reviews by [4,9-11]).

Both inducible and constitutive costs may play a role in maintaining polymorphism for resistance to infection. Specifically, host genotypes enduring costs (either because they launch powerful, self-damaging responses, or because they have invested heavily in preparatory defences) may be outcompeted when the threat of parasitism recedes. A great number of studies, particularly in invertebrates, have tested for the presence of both forms of costs of resistance. Costs are clearly present in some systems (Table 1 [reviewed in [12,13]), but not in others, where they may be transient or manifest only in a subset of life history traits. Some examples of cost-free resistance appear certain. For example, more than 100 years after their introduction outside the range of their natural parasite, *Microphallus sp*, an experimental study found that *Potamopyrgus *snails are still resistant, which would not be expected if resistance bore fitness costs [14]. Costs are almost certainly not universal.

**Table 1:** Literature survey of studies testing for constitutive (a) and inducible (b) costs of immunity in arthropods.

Ref	Species	Cost ?	Selection	Challenge	Environment	Observations
a Constitutive cost						
[35]	*Plodia interpunctella*	**development time^a^**, longevity, reproduction	resistance virus	-	Standard lab conditions	^a^development time difference tend to decrease after two more generations
[41]	*Plutella xylostella*	growth rate^b^, survival^b^	resistance *B. thuringiensis*	-	Poor quality food	^b^cost of resistance for one population, advantage for the other
[41]	*Plutella xylostella*	survival^c^	resistance *B. thuringiensis*	-	larval competition	^c^cost of resistance for one population on two tested
[55]	*Biomphalaria glabrata*	**fertility**, mortality	resistance *Schistosoma*	-	Standard lab conditions	includes a susceptiblity-selected control (different effect)
[34]	*Aedes aegypti*	early pupation = low melanization	early/late pupation	beads to test melanization	Standard lab conditions	no control for wounding
[6]	*Tenebrio molitor*	cuticular color^d^	-	-	Standard lab conditions	^d^cuticular color is correlated with investment in immunity; positively correlated with longevity, no effect on fecundity
[56]	*Anopheles gambiae*	longevity, fecundity, mating success	resistance *Plasmodium*	-	Standard lab conditions	includes a susceptiblity-selected control (same effect)
[36]	*Drosophila melanogaster*	**competitive ability against controls^e^**, survival, development time, fecundity, size, fluctuating asymetry	resistance *A. tabida*	-	Various food levels and competition	^e^cost found only for lowest food levels
[37]	*Drosophila melanogaster*	fecundity, egg viability, starvation tolerance	resistance *L. boulardi*	-	Standard lab conditions	
[37]	*Drosophila melanogaster*	competitive ability^f^	resistance *L. boulardi*	-	Various food levels and competition	^f^cost found only for lowest food levels
[55]	*Drosophila melanogaster*	resistance, survival, competition ability^g^	high/low densities	-	Standard lab conditions	^g^individuals selected at high densities fare actually better
[57]	*Drosophila melanogaster*	**fecundity^h^**, longevity	resistance *Macrocheles*	-	High/Low temperatures	^h^cost found only for high temperature
[58]	*Drosophila melanogaster*	fecundity	-	-	Standard lab conditions	
[8]	*Drosophila melanogaster*	fecundity^i^	-	-	High/Low food levels	^i^cost found only for low food
[59]	*Drosophila melanogaster*	fecundity^j^, competitivity^j^	resistance *T. kingi*	-	High/Low larval competition	^j^cost found only for high competition level
[39]	*Drosophila melanogaster*	**longevity**^k^, body mass, development time^l^**, egg viability**, productivity, mating	resistance *P. aeruginosa*	-	Standard lab conditions	^k^cost found only for females; ^l^reduced development time for selected lines
[60]	*Acyrthosiphon pisum*	fecundity, resistance to different parasites	-	-	High/low food quality	
[53]	*Acyrthosiphon pisum*	**fecundity**, survival without food^m^, size^m^, competitive ability^n^	-	-	Standard lab conditions	^m^resistance is actually positively correlated with this trait; ^n^unpublished data cited
[32]	*Daphnia magna*	mortality, age of 1st reproduction, fecundity	-	-	Standard lab conditions	
[32]	*Daphnia magna*	competitive ability	-	-	High densities, very low food	
[31]	*Daphnia magna*	fitness	-	-	Low quality natural environment	
[33]	*Daphnia magna*	survival, fitness	+/- *O. bayeri*	-	low density, high food	
**b Inducible cost**						
[40]	*Tenebrio molitor*	**?^o^**	-	generalist fungus	solitary/gregarious	^o^assume plasticity = cost
[6]	*Tenebrio molitor*	**longevity**, fecundity	-	Nylon inserted	Standard lab conditions	
[61]	*Tribolium castaneum*	fecundity; **development^p^, survival^p^**	-	heat killed bact injected	Standard lab conditions	^p^costs shown in 1 line only
[62]	*Bombus terrestris*^q^	**survival^r^**	-	LPS injected, beads inserted	High/zero food levels	^q^sterile workers; ^r^cost found only when starved
[63]	*Aedes aegypti*	**fecundity^s^**	-	beads inserted	Standard lab conditions	^s^depend on the charge of the bead
[8]	*Drosophila melanogaster*	**fecundity^t^**	-	*P. rettgeri *injected	High/low food levels	^t^costs result from wounding only, just after injection (not lasting)
[59]	*Drosophila melanogaster*	**fecundity**, survival	resistance *T. kingi*	exposure *T. kingi*	Standard lab conditions	
[25]	*Daphnia magna*	**mortality**	-	exposure *P. ramosa*	low food and high density^u^	^u^high density for first experiment only
[31]	*Daphnia magna*	fitness	-	exposure *O. bayeri*	Low quality natural environment	

Details given for each study are: the species studied (Species), whether a cost was found or not (Costs?: traits for which a cost was detected are bolded), whether the lines used in the experiment where selected before, and if so the object of the selection (Selection), how the animal's immunity was challenged (Challenge) and finally what were the environmental conditions tested (Environment).

*Daphnia magna*, a planktonic crustacean found in temperate freshwater ponds, has been the object of considerable research regarding parasitism [reviewed in [15]. Substantial genotypic variability for resistance has been found in natural populations [15-19], while studies incorporating environmental variation (temperature and food levels) have found pervasive genotype-by-environment interactions, indicating that the environment may change the fitness consequences of parasitism [20-24]. Past studies on costs of resistance in *Daphnia magna *indicated no constitutive costs, but detectable costs of launching an immune response (Table 1[25]). We sought to extend understanding of the costs of launching an immune response by testing if costs were enhanced with successive exposures to the parasites, or under certain (harsh) environmental conditions. This investigation of induced costs of exposure yielded results that did not agree with previous work [25]. In an effort to settle the issue, we gathered additional data sets that were originally produced for other questions, but which were suitably designed such that costs of induced immunity could be probed. In sum, we present the results of five experiments to show that, under a variety of genetic backgrounds, exposure protocols, and environmental conditions, costs of immunity are only occasionally detected in the *D. magna-P. ramosa *system.

## Methods

Below, we describe the detailed methods of the three main experiments, which are new experiments designed to test for costs of resistance. For brevity, we only report the results and a tabular summary of methods for the additional experiments (i.e. those which, although not originally designed for testing costs, could nevertheless be used for that purpose). The detailed methods for the set of additional experiments are reported in the Additional file 1. However, the following descriptions of host and parasite biology, as well as the general experimental schemes, are applicable to all experiments reported.

### *Daphnia magna *clones and *Pasteuria ramosa *strains

*Daphnia magna *is a filter-feeding crustacean zooplankter that reproduces by cyclical parthenogenesis. *Pasteuria ramosa *is a gram-positive bacterium that is an obligate, spore-forming endoparasite of *D. magna*. Hosts become infected with *P. ramosa *by filtering transmission spores present in the water or sediments at the pond bottom. Infection causes host castration and gigantism, as well as premature death. Within the host, *P. ramosa *goes through a developmental process that culminates in the formation of spores. Host death is essential for transmission, mature spores being released from the remains of dead infected hosts. *P. ramosa *spores are horizontally transmitted only, i.e. there is no evidence of transovarial infection [26].

In the main experiments, we used 4 *Daphnia *clones, named GG3, GG4, GG7 and GG13, which were originally collected in Germany from a population near Gaazerfeld [16]. Two strains of the parasite *P. ramosa *were used, Sp1 and Sp8; they each originated from the same Gaarzerfeld population and have been used to infect *Daphnia *in the laboratory for over a decade (they were originally named 1 and 8 [16]). The *Daphnia *hosts were maintained in the laboratory in a state of clonal reproduction, whereas the *P. ramosa *strains were kept frozen until needed for the experiments. From Carius et al. [16], we know that GG3 and GG4 are relatively susceptible clones to a variety of parasite strains, whereas GG7 and GG13 are mostly resistant. Similarly, Sp1 is a relatively highly infective *P. ramosa *strain, whereas Sp8 is comparatively innocuous. The specific infection levels expected for the different host-parasite pairs are indicated in Table A3 (Additional file 1).

### General experimental protocol

To equilibrate maternal effects prior to the experiments, replicate jars of each host clone were kept under controlled conditions for three generations: 20°C (in temperature-controlled incubators), a set light:dark cycle (Experiment 1: 12:12; Experiments 2 & 3: 16:8 hours), and fed equal amounts of chemostat grown algae (*Chlorella *sp. or *Scenedesmus *sp., see Table 2) per *Daphnia *per day (quantity varies among experiments, see Table 3). Replicates contained 5 females, either in a 60 ml or 200 mL jar of *Daphnia *medium, depending on the experiment (Aachener Daphnien Medium [27], see Table 2). Medium was changed every 2 to 3 days.

**Table 2:** Details of experimental designs presented in the current study.

			Exposure					Food						
										
#	Host clones	Parasite strains	spore nb	nb	time (days)	age (days)	Jar size (mL)	**Sp**.	qty (×10^6^)	T°(°C)	rep	ind/jar	**Cont**.	Expe time (Days)
1	GG3,GG4, GG7,GG13	Sp1,Sp8	50000	1	2	5	60	*C*	3.5	20	35	1	c. *D*.	last death
													
		Sp1	200000											

2	GG4,GG7	Sp1	20000	1, 2, 4	2	5, 11, 19, 27	60	*C*	3.5	20	40	1	c. *D*.	last death

3	GG4	Sp1	2500	1, 2	10^a^	1, 5^a^	60	*C*	2.1	15, 20,25	30	1	c. *D*. H_2_O	60 days^a^

4	GG3	Sp1	5000	1	5	1	60	*S*	5	20	24/72	1	c. *D*.	38 days
														
			50000											
														
5	<20 clones, recently wild-caught^b^	Mix wild spores	50000	1	5	1	200	*S*	5	20	70	5	-	35
														
			100000											

^a^at 20°C, degree-day equivalent for other T°

^b ^Hosts were collected from a Scottish population in Summer 2003, see Duncan and Little *Evolution *2007, 61(4):796-803

For each experiment (referred to by their number: #), the *D. magna *clones (Host clones) and the *P. ramosa *strains (Parasite strains) used are indicated. The protocol used for the exposure(s) is given, described by the number of spores per *Daphnia *added (spore nb), the number (nb), the length (time) and the age (age) of the individuals when each exposure was performed. The environmental conditions of the experiment are detailed. For food, the algae species (Sp.: *C *for *Chlorella, S *for *Scenedesmus*) and the quantity (qty, in millions of cells) are indicated. The temperature(s) at which the experiment was performed (T°), the number of replicates per treatment (rep), the number of individuals per jar (ind/jar), the jar size, the controls used [Cont.: crushed unexposed *Daphnia *(c. *D*.) or sterile water (H_2_O)] and the total length of the experiment (Expe time) are also given.

**Table 3:** Costs in *Daphnia magna*.

#	Inducible Cost?	Challenge	Environment	Observations
1	age first reproduction, size, **survival^a^**, **fecundity^a^**	various strains and doses	low food	^a ^only for one *Daphnia *clone (GG3)/*Pasteuria *strain combination at high dose

2	age first reproduction, survival, fecundity	multiple exposures	low food	

3	fecundity, survival	single or double dose	various T°, low food	

4	fecundity, **survival**	single or double dose	normal	one *Daphnia *clone only (GG3)

5	**survival**	single or double dose (no control)	normal	many genotypes (wild-caught *Daphnia*)

For each experiment (referred to by their number: #), the finding (bolded) or absence (normal) of inducible costs of immunity in response to exposure is indicated for the traits tested. Details of the type of challenge and experimental environment for each experiment are also given.

For the parasite exposures, we used a split-brood design: offspring (less than 24 hours old) of each replicate jar were split into the different treatments (various spore types, *D. magna *clones, spores quantity, number of exposures, temperatures, see Table 3 and Additional file 1 S1). On the day of exposure, the medium was changed, a teaspoon of sterile sand and a solution of *P. ramosa *spores (treatments) or a sham solution (controls) were added to each jar. Exposure length was variable among experiments (Table 2). During the exposure, the sand was stirred daily, and the *Daphnia *were fed with low amounts of food (Table 2). The combination of sand and low food increases bottom grazing behaviour, thus increasing the chances of *Daphnia *encountering the parasite spores.

After the exposure period, all *Daphnia *were transferred to new jars with new medium. The medium was changed when hosts produced a clutch, or every 2 or 3 days if they did not. The recording of infection status began from 10 to 16 days after exposure: by this time infected individuals were red in colour, larger and had mostly ceased reproducing. Individuals that died before infection assessment were removed from the analyses. The presence of a clutch was checked for daily or every other day (depending on the experiment, see Additional file 1 S1), and the number of offspring and clutch date were recorded. When an individual was dead, the death date was recorded and the individual was transferred into a 1.5 ml Eppendorf tube, dried and frozen at -20°C. Frozen *Daphnia *were later crushed in CASY^®^ton solution and *P. ramosa *transmission stages were counted using a CASY^® ^model DT electronic cell counter (Innovatis AG). The total experiment length was variable among experiments (see Table 2). Tables 2 and 3 provide a summary of the protocols and treatments used in the different experiments. Additional details of each experiment can be found in Additional file 1 (A1).

### Statistical analyses

To test the effect of the treatment on the proportion of hosts that became infected, we used generalized linear models of the form: PROPORTIONINFECTED = GENO*EXPO (main effects and 2-way interactions), where the response variable PROPORTIONINFECTED is a proportion (binomial error) and the explanatory variables are categorical: GENO is the *Daphnia *genotype (clone) used (number of levels equal to number of genotypes) and EXPO is the type of treatment (number of levels equal to number of treatments). Genotype is a fixed effect rather than a random effect because it is both replicated and we were interested in clonal means. Models were checked for overdispersion. For other traits, we used general linear models (GLM) of the form: TRAIT = GENO*INF*EXPO (main effects, 2-ways and 3-ways interactions), where the response variable TRAIT is continuous (normal error) and INF is the infectious status (categorical, 2 levels: infected or uninfected). Normality of residuals was checked and the data were log-transformed when necessary to ensure normality. For analyses of age of first reproduction and for age of death (survival) we also used Cox's proportional hazards models (CoxPH) of the form TRAIT = GENO*EXPO*INF. Hosts that did not die by the end of the experiment were entered as censored data. For Experiment 3 (Table 2), the degree-day was used as the time scale to allow comparisons between temperature treatments; this is the product of the real day by the temperature, and is used as an approximate measure of *Daphnia *physiological time [22].

The models were simplified according to Crawley [28]: significance of the different terms was tested starting from the higher-order terms. Non-significant terms (*P *> 0.05) were removed. When an interaction term was significant, each level of the factors in the interaction were then analysed separately. Factor levels of qualitative variables that were not different in their estimates were grouped, as described by Crawley [28]. This process gives the minimal model. Analyses were performed using the R freeware package (v 2.0.1, http://www.r-project.org; Experiments 1, 2, 4 and 5) or the JMP 7 (SAS Institute Inc., Experiment 3).

## Results

A results summary is provided in Table 3, indicating whether evidence for costs of resistance was detected or not. As this study aims at identifying potential costs of resisting parasites, only data from uninfected hosts are presented, which includes both unexposed individuals (controls) and exposed but not-infected individuals (i.e. individuals that resisted the infection). The results on costs of parasitism (i.e. the effects of infection on life history traits for exposed and infected individuals) are presented in Additional file 1 (A2).

### Experiment 1: single exposure, four clones

**Figure 1:**
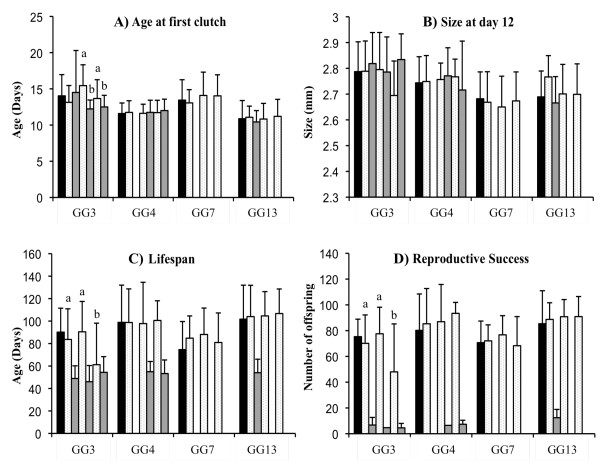
**Experiment 1 results (four *D. magna *host genotypes exposed once to *Pasteuria ramosa*, see text)**. In all panels, the black bar represents means for unexposed controls. For the exposed individuals, light grey bars are means for uninfected hosts and dark grey bars are means for the infected. For each genotype, the exposure order is always: controls, parasite spore 8 (Sp8) low dose (uninfected/infected), parasite spore 1 (Sp1) low dose (uninfected/infected) and Sp1 high dose (uninfected/infected). Panel A presents for each category the average age at which females released their first clutch (for GG3, *a *and *b *represent two statistically different groups, see text). Panel B presents for each category the average body size of host at day 12. Panel C presents for each category the average age on the day of death (for GG3, *a *and *b *represent two statistically different groups, see text). Panel D presents for each category the mean reproductive success, i.e. the number of offspring produced during the entire life (for GG3, *a *and *b *represent two statistically different groups, see text). Bars are standard errors.

In this experiment, we exposed four clones of *Daphnia *with relatively extreme resistance phenotypes (GG3 and GG4 are generally susceptible (S), while GG7 and GG13 are generally resistant (R) [16]) to different strains and doses of *P. ramosa *(Table 2). For a total of 560 exposed individuals, we report measurements of four life history traits: age at first reproduction, size (body length measured for each individual on day 12, see Additional file 1), lifespan and lifetime number of offspring (Figure 1). We were thus able to examine inducible costs of resistance, by comparing between unexposed and exposed-but-uninfected individuals. Note that for survival, only the GLM analyses are reported, as the CoxPH analyses led to identical results. The rates of infection observed in this experiment for the various host clones and parasite strains were similar to those expected from an earlier study (Additional file 1, Table A3). We found a significant effect of the genotype for the age at first reproduction (GENO: *F *= 47.7, *P *< 0.001, Figure 1A), for size (GENO: *F *= 20.37, *P *< 0.001, Figure 1B), for lifespan (GENO: *F *= 16.12, *P *< 0.001, Figure 1C) and for the total number of offspring (GENO: *F *= 2.75, *P *< 0.05, Figure 1C). However, while this shows that there are genotypic determinants for performance, they do not appear to be related to resistance phenotypes.

Induced costs of resistance are indicated when the performance of exposed-but-uninfected individuals is poorer than that of unexposed controls. For age of first reproduction, there was no difference between controls and exposed-uninfected individuals for any clone, regardless of exposure dose (GENO:EXPO: *F *= 0.72, *P *= 0.69; EXPO: *F *= 0.97, *P *= 0.40). For body size, the 3-way interaction Geno:INF:EXPO was significant(*F *= 4.93, *P *= 0.026), but we found no clear effect of exposure (EXPO: *F *= 0.36, *P *= 0.78), all treatment sizes being globally similar to that of controls. The significance of the 3-way interaction is probably due to GG3 uninfected individuals exposed to high dose of Sp1 being smaller than expected, and GG13 uninfected individuals exposed to Sp8 being larger than expected (Figure 1B). For the age of death, we found no effect of exposure, except for GG3 individuals exposed to high dose of Sp1, for which lifespan is shorter than controls (*F *= 3.53, *P *= 0.019; Figure 1C). Finally for the total number of offspring produced during the individuals' life, again only GG3 individuals exposed to a high dose of Sp1 showed significantly lower fecundity than controls (*F *= 4.83, *P *= 0.004; Figure 1D). The analysis of other fecundity related traits (number of clutches, average clutch size, data not shown) indicates that this lower fecundity is essentially due to a reduction of the clutch number, which is probably linked to the reduction of their lifespan.

### Experiment 2: multiple exposures, two clones

**Figure 2:**
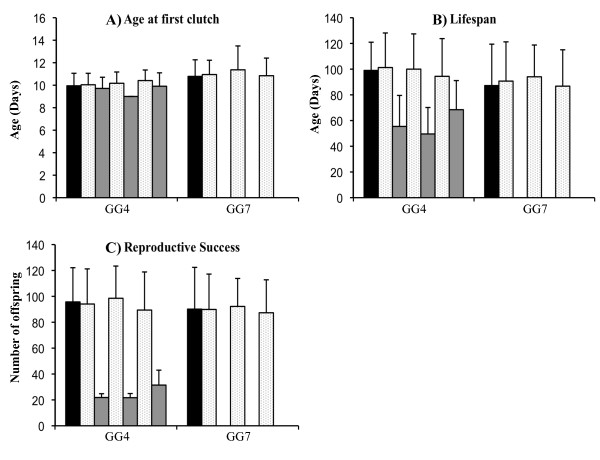
**Experiment 2 results (two host genotypes and multiple parasite exposures, see text)**. The black bar represents means for unexposed controls. For the exposed individuals, light grey bars are means for uninfected and dark grey bars are means for infected. For each genotype, the exposure order is always controls, single exposure (uninfected/infected), double exposure (uninfected/infected) and quadruple exposure (uninfected/infected). Panel A presents for each category the average age at which females released their first clutch. Panel B presents for each category the average age on the day of death. Panel C presents for each category the mean reproductive success i.e. the number of offspring produced during the entire lifetime. Bars are standard errors.

In this experiment, we tested for inducible costs of resistance, by applying multiple parasite exposures. The assumption is that repeated exposures lead to repeated, and thus more costly, immune responses. Two host clones (GG4 (S) and GG7 (R)) were exposed to spores of *P. ramosa *strain Sp1. Three exposure treatments were carried out, with hosts being exposed either once, twice or four times to 20,000 spores each time (with a week between exposures). A total of 320 individuals were analysed for the same traits as in Experiment 1 (Figure 2). Again, the GLM and CoxPH analyses are similar, so only the GLM is reported. Rates of infection observed in this experiment were lower than that observed in Experiment 1, as expected due to the lower spore dose used (for more details see Additional file 1 A2). In comparing the performances of exposed-but-uninfected individuals to controls, we found no effect of the number of exposures either for the age of first reproduction (EXPO, *F *= 0.64, *P *= 0.59, Figure 2A), the time to death (EXPO, *F *= 0.64, *P *= 0.59, Figure 2B) or for the total number of offspring (EXPO, *F *= 0.68, *P *= 0.56, Figure 2C). However, we found a genotype effect in each case, confirming Experiment 1: GG4 individuals reproduce earlier (GENO, *F *= 8.33, *P *= 0.004), die later (GENO, *F *= 7.39, *P *= 0.007) and reproduce more (GENO, *F *= 9.06, *P *= 0.003) than GG7 individuals.

### Experiment 3: One or two exposures, three temperatures, one clone

Given recent research on the environment-dependent nature of infection outcomes in host-parasite systems [13], we performed this experiment to test whether costs of resisting infection were temperature dependent. We chose a single host clone (GG4) and parasite strain (Sp1) and exposed a total of 360 individual *Daphnia *to either single or double doses (2,500 spores per dose) of *P. ramosa *at 15°C, 20°C, and 25°C (Table 2). No significant difference was detected between our two control treatments (ddH2O or healthy crushed *Daphnia*) for either fecundity (*F *= 0.316, *P *= 0.576) or lifespan (*χ^2 ^*= 0.212, *P *= 0.645), therefore we combined these into one 'control' treatment.

**Figure 3:**
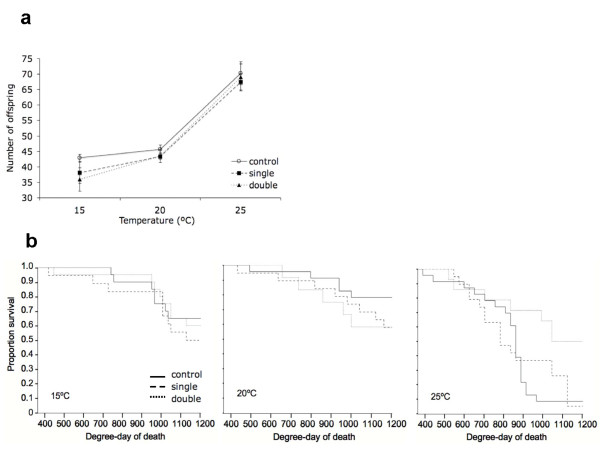
**Experiment 3 results (one or two exposures, three temperatures, but only one host genotype, see text)**. Panel A presents the average number of offspring produced per *Daphnia *until degree-day 700 at 15°C, 20°C, and 25°C. Panel B presents the proportion of hosts alive until degree-day1200 at 15°C, 20°C, and 25°. For panels A and B, full black lines represent hosts that were not exposed to parasites, dashed lines and dotted lines those that were exposed to a single or a double dose (respectively) but did not develop infection. Bars are standard errors.

Again, we measured the cost of resisting infection (i.e. inducible cost) as the reduction in either fecundity or survival in hosts that were exposed to parasites but did not develop infection, relative to unexposed controls. We found a significant main effect of temperature on fecundity (*F *= 89.11, *P *< 0.001) and lifespan (*χ^2 ^*= 23.86, *P *< 0.001), but no effect of dose nor a dose-by-temperature interaction for either trait (DOSE: fecundity: *F *= 1.41 *P *= 0.247; lifespan *χ^2 ^*= 2.65 *P *= 0.266; DOSE:TEMP fecundity: *F *= 0.30 *P *= 0.878; lifespan *χ^2 ^*= 9.41 *P *= 0.052; CoxPH analyses yield the same results; Figure 3). This suggests that while temperature affects the expression of these life-history traits, being exposed or not to *P. ramosa *had little or no effect, and this was the case at all temperatures.

### Additional datasets

We analysed two additional datasets for inducible costs of resistance where exposed but uninfected hosts could be compared with unexposed hosts.

#### Experiment 4

**Figure 4:**
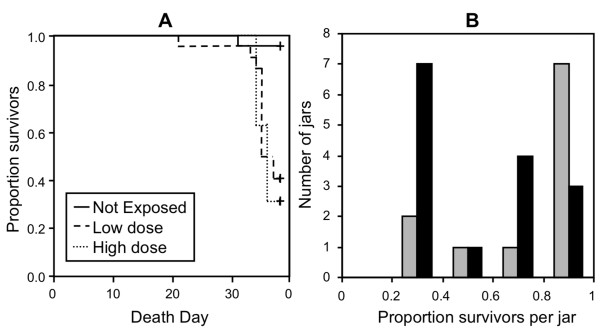
**Experiments 4 and 5 results**. Panel A presents the results of experiment 4: survival curves of uninfected individuals are presented for different exposure doses. Panel B presents the results of experiment 5: the distributions of the proportion of live uninfected individuals per jar after 35 days are presented for two doses of parasites (50, 000 spores, grey bars; 100, 000 spores, dark bars).

In this experiment, we gathered data from a 38-day survey of survival and fecundity traits in *D. magna *genotype GG3 either exposed to two doses (5,000 or 50,000 spores per *Daphnia*) of *P. ramosa *(Sp1) or not-exposed. Among the exposed individuals, we only considered those not infected (low dose: 39 individuals out of 72, high dose: 21 out of 72). We compared them with unexposed controls (*N *= 23) for several life history traits. We found no effect of exposure for the number of offspring (EXPO: *F *= 0.17, *P *= 0.85) or the number of clutches (EXPO: *F *= 1.35, *P *= 0.26; data not shown). Regarding lifespan (Figure 4A), 22, 11 and 8 individuals were still alive at the end of the experiment respectively for not-exposed, low dose and high dose treatments). A CoxPH model with censorship indicated exposed individuals died significantly more than those not exposed (Likelihood Ratio Test (LRT) = 30.6, *P *< 0.001).

#### Experiment 5

We used data from *Daphnia *exposed to one of two doses (50,000 and 100,000 spores per *Daphnia*) of *P. ramosa *(the spore solution was a mix of spores collected from the same pond as the *Daphnia*, see Additional file 1 A1). We only included in the analysis replicate jars where none of the 5 individuals were infected (26 jars out of 141). We found no significant effect of exposure for the mean clutch size (EXPO: *F *= 4.12, *P *= 0.054; data not shown). However, hosts exposed to 100, 000 spores died at a faster rate compared to those exposed to 50, 000 spores (EXPO: LRT = 8.86, *P *= 0.003; Figure 4B).

## Discussion

Investing in immune defence is thought to bear fitness costs in the absence of infection, as investing in preventing or fighting infection should divert resources from other fitness-related traits [e.g. [4,6-8,29,30]]. Consequently, the fittest genotype should not necessarily be the most resistant; it will be the one with optimal investment in the various fitness traits in a given environment. Two types of costs have been widely studied: constitutive costs (i.e. the cost of being resistant in absence of parasites) and inducible costs (i.e. the cost of using the immune system when challenged by a parasite). Table 1 presents a (non-exhaustive) survey of studies (*N *= 24; note that some publications describe several independent studies, which are thus presented individually), which have looked for one or the other type of cost. Although there may be a publishing bias toward studies demonstrating costs, Table 1 indicates that costs of immunity are not uncommon: 12 studies out of 22 found evidence of a constitutive cost of resistance for at least one of the life history traits measured, and for inducible costs of immunity, eight studies of nine documented them.

To this list, we now add five additional experiments based on the *D. magna-P. ramosa *interaction. The first experiment investigated inducible costs of resistance with different *P. ramosa *strains and doses on 4 *Daphnia *clones (Figure 1). There were no general fecundity or survival costs of being exposed to the parasite, except perhaps for one highly susceptible host clone (GG3) which showed delayed development, lower reproductive success and shorter lifespan when exposed to the highest dose of Sp1 (the most virulent parasite strain). A second experiment expanded this work by applying repeated exposures (under the assumption that this would be more costly to resist, Figure 2), while a third tested if costs might be more evident under temperature stress (Figure 3), but none of these experiments yielded measurable costs. Finally, we analysed two additional datasets that were appropriate for testing for inducible costs. These experiments (experiments 4 and 5) both revealed that the individuals exposed to a higher parasite spore dose died faster than those exposed to lower quantity of parasite spores or not exposed (Figure 4). These last results are similar to a previously reported one [25] where higher exposure doses also induced high mortality amongst host that fought off infection.

Thus, while inducible costs of resistance are occasionally detectable, they clearly are not as pervasive in the *Daphnia-Pasteuria *interaction as they appear to be in other systems (Tables 1 and 3[31]). Similarly, constitutive costs have not been detected in *Daphnia*, either towards *P. ramosa *[32] or other parasites [31,33]. It would appear that both types of costs of resistance to parasitism in *Daphnia *are at best elusive and condition-dependent, and might be of little evolutionary relevance.

It is difficult to say at present why costs are more prevalent in some systems, and it may simply be that the various host-parasite systems have different evolutionary histories; some of these lead to costs, others do not. However, we wish draw attention to three aspects related to experimental design. First, some studies documenting costs used lines that were artificially selected for resistance to a particular parasite or for a life-history trait modification (e.g. early or late pupation[34-36]), and there are a number of reasons why such studies could misrepresent the importance or pervasiveness of costs in natural settings. For example, deleterious mutations can hitchhike with resistance [13,37,38], leading to an overestimation of the magnitude of costs. Second, many studies of cost used artificial rather than natural host/parasite combinations [e.g [39,40] and/or artificial immune stimulation (injection, beads insertions, see Table 1[13,33,37,38,41]. It is expected that while new parasite challenges induce costly responses, in a longer-term, coevolving interaction, the response is possibly more finely tuned (e.g. more specific [42]), and will carry little cost [43]. This is illustrated by a study on the isopod *Asellus aquaticus*, which displays costly responses to an acanthocephalan parasite in naïve populations where the parasite is unknown, but resistance appears to be cost-free in coevolving populations [44]. A third critical point about documented immunity costs is that they are mostly detected in quite extreme conditions (low food, high densities, or extreme temperatures; Table 1 [e.g. [12]]), which may differ from those used during selection for resistance [33]. While it is often assumed that harsh conditions actually reveal the existence of a cost [e.g. [12]], the evolutionary significance of such costs in natural conditions may be debateable [13,33,41,42,45]. The three main experiments presented here were carried out under relatively low food quantities, as a shortage of food is thought to be a stressor that can reveal costs [8,12,36,37]. In addition, experiment 3 included a temperature treatment of 25°C, a stressful temperature at which host physiology is suboptimal [46] and background mortality in increased [21,24], and yet costs of resistance remained undetectable.

Costs can potentially contribute to the maintenance of resistance polymorphism in host-parasite interactions [4,7,30,47,48], but depending on the nature of the genetic variation that underlies susceptibility, costs may not be needed to maintain polymorphism [49,50]. Specifically, under a "gene-for-gene" model of genetic specificity, where a mutation in the host allows resistance to any genotype of the parasite, the resulting dynamic is asymmetrically frequency dependent (i.e. repeated selective sweeps of universally infective parasite strains), and costs are needed to prevent fixation of host resistance [49]. By contrast, under a "matching-allele" model [51] of genetic specificity, resistance requires an allele that matches the parasite virulence allele. In this case, the host is resistant to that genotype of parasite, but remains susceptible to the others, which results in symmetrical frequency dependence, where resistance costs are not needed to maintain susceptibility genotypes [49]. The presence of strong genotype-by-genotype interactions in the *D. magna-P. ramosa *system, coupled with a lack of apparent costs, supports a "matching-allele" coevolution scenario in this system [16].

## Conclusions

Moving beyond simple genetic models, complex immune systems may incorporate substantial redundancy [13,43] to face the changing challenges and selection pressures in a dynamic environment [52]. Thus, evaluating costs probably requires detailed mechanistic and genetic knowledge about resistance to actually measure the pleiotropic effects of a single modification, instead of a general phenotypic effect incorporating multiple effects that potentially compensate each other [43,50]. Still, the now extensive work on the *D. magna-P. ramosa *does not appear to suggest a crucial role of immunity costs in their coevolution [14,31,33,44]. Other studies have even documented advantages rather than costs linked with constitutive resistance in absence of parasite (increased survival [6,41,53,54], competitive ability [54] or reduced development time [39]), suggesting a limited role for costs in coevolution, or that more complex processes are at work.

## Authors' contributions

PL designed, carried out and analyzed experiments 1 and 2, and drafted the manuscript. PV designed, carried out and analyzed experiment 3, and helped to draft the manuscript. TL participated in the design of the study, analyzed the data for experiments 4 and 5, and helped to draft the manuscript. All authors read and approved the final manuscript.

## Supplementary Material

Additional file 1 **Experimental details and infection costs.** A1: Detailed experiments protocols. A2: Analyses of infection costs. Table A3: Infection levels for the different host-parasite combinations. Fig. A4: Experiment 2 spore loads. Fig. A5: Experiment 3 spore loads. Click here for file
