# Supplementary material for: Successfully resisting a pathogen is rarely costly in Daphnia magna
Source: BMC Evol Biol. 2010 Nov 17;10:355. doi: 10.1186/1471-2148-10-355 (PMC2998533; doi:10.1186/1471-2148-10-355)
Supplement: Additional file 1 — Experimental details and infection costs. A1: Detailed experiments protocols. A2: Analyses of infection costs. Table A3: Infection levels for the different host-parasite combinations. Fig. A4: Experiment 2 spore loads. Fig. A5: Experiment 3 spore loads. [file 1471-2148-10-355-S1.DOC]

**Successfully resisting a pathogen is rarely costly in *Daphnia magna***

Additional file 1

**A1: Experiments protocols**

***Experiment 1: Single exposure, four clones***

This experiment was set up to assess the effect of parasite exposure on host fitness. We used the four *Daphnia* clones (GG3 and GG4, both relatively susceptible, and GG7 and GG13, both relatively resistant) and the two parasite strains (Sp1, highly infective, and Sp8, lowly infective). To equilibrate maternal effects prior to the experiment, 35 replicates of each host clone were kept under controlled conditions for three generations. These controlled conditions were 20°C (in temperature-controlled incubators), a light:dark cycle of 16:8 hours, and 3.5 x10^6^ cells of chemostat grown algae (*Chlorella* sp.) per *Daphnia* per day. It is important to note that this quantity of food is relatively low, half of what is standard for *Daphnia* to thrive in our laboratory. Replicates contained 4 females, in a 60ml jar of *Daphnia* medium. All subsequent generations of each replicate, including the experimental generation, were seeded using females from the 3^rd^ or 4^th^ clutches that were less than 24 hours old.

For the experiment, 4 offspring (less than 24 hours old) from each of the 35 replicate jars were put individually in a jar containing 60mL of *Daphnia* medium (thus 4 jars with one *Daphnia* per replicate; Day 1). On day five, medium was changed and half a tea-spoon of sterile sand was added to each jar (the addition of sand and the low food both increase bottom grazing behaviour, which increases the chances of *Daphnia* encountering the parasite spores and reduces variation among individual exposure). The four jars of each replicate were each submitted to a different treatment: a) the first jar received a control solution consisting of crushed unexposed adult *Daphnia* (“unexposed” treatment); the other jars received a parasite spore solution consisting of crushed infected adult *Daphnia*: b) one jar received a solution with ~50,000 Sp8 spores (“Sp8 low dose” treatment), c) one jar received a solution with ~50,000 Sp1 spores (“Sp1 low dose” treatment) and d) the last jar received a solution with ~200,000 Sp1 spores (“Sp1 high dose” treatment). The treatment lasted 48 hours, the sand being stirred just after treatment start and 24 hours later. A total of 560 individuals were thus treated. After the treatment period, all individual *Daphnia* were transferred to new jars with 60mL of new medium and kept in normal controlled conditions. Experiment individuals were fed with 1.75 x10^6^ algae per day for the first two days after hatching and then 3.5 x10^6^ algae per day until their death (including during the infection period). The medium was change every 2 days. The experiment lasted until the death of the last individual.

On day 12, all individuals were measured: a picture was taken under binocular, with the same magnification for all; the size was then measured on photo using the Mesures v1.0 module (P. Flocard) of the Photofiltre v6.3.1 freeware (A. Da Cruz, http://photofiltre.free.fr). Each jar was checked daily. When newborn were present, the adult females were moved to a new jar, and the offsprings counted and the date of hatching recorded. When an individual was dead, the death date was recorded and the individual was transferred into a 1.5ml Eppendorf tube, dried and frozen at -20°C. Frozen *Daphnia* were later crushed in 500 µL of CASY®ton solution and *P. ramosa* transmission stages were counted in a 50μl sample of this solution using a CASY® model DT electronic cell counter (Innovatis AG). Infection status was recorded 16 days after exposure (Day 23): by this time infected individuals were red in colour, larger and had mostly ceased reproducing (*P. ramosa* castrates its host). Individuals dead before infection assessment were removed from the analyses.

***Experiment 2: Multiple exposures, two clones***

To assess the effect of multiple parasite exposures on host fitness, we used only two host clones (GG4, relatively susceptible, and GG7, relatively resistant) and one parasite strain (Sp1, highly infective). The rearing conditions were similar to experiment 2 (20°C, of 16:8 hours light:dark cycle, 3.5 x10^6^ food algae per *Daphnia* per day). Three generations of similar controlled conditions were applied to 40 replicates of each host clone prior to the experiment to equilibrate maternal effects.

For the experiment, 4 offsprings (less than 24 hours old) from each of the 40 replicate jars were put individually in a jar containing 60mL of *Daphnia* medium (thus 4 jars with one *Daphnia* per replicate; Day 1). Two treatment solutions were prepared, one control solution consisting of crushed unexposed adult *Daphnia* and one spore solution consisting of crushed Sp1 infected adult *Daphnia*; one volume of the spore solution correspond to ~20,000 Sp1 spores (we used a low dose as Sp1 is highly infective, and we wanted to get some susceptible individuals to remain uninfected at the end of the experiment). The four jars of each replicate were each submitted to a different treatment on day 5, 11, 19 and 27: a) the first jar received each time one volume of the control solution (“unexposed” treatment); b) the second jar received one volume of the spore solution on day 5 and then 1 volume of the control solution the other days (“single exposure” treatment), c) the third jar received one volume of the spore solution on days 5 and 11 and then 1 volume of the control solution on days 19 and 27 (“double exposure” treatment) and d) the last jar received each time one volume of the spore solution (“quadruple exposure” treatment). On each day of treatment, the medium was changed and half a tea-spoon of sterile sand was added to each jar. Each treatment lasted 48 hours, the sand being stirred just after treatment start and 24 hours later. A total of 320 individuals were thus treated. After each treatment period, all individual *Daphnia* were transferred to new jars with 60mL of new medium and kept in normal controlled conditions. Experiment individuals were fed with 1.75 x10^6^ algae per day for the first two days after hatching and then 3.5 x10^6^ algae per day until their death (including during the infection periods). The medium was change every 2 days. The experiment lasted until the death of the last individual.

Each jar was checked every other day. When newborn were present, the adult females were moved to a new jar, and the offspring counted and the date of hatching recorded. When an individual was dead, the death date was recorded and the individual was transferred into a 1.5ml Eppendorf tube, dried and frozen at -20°C. *P. ramosa* transmission stages were counted in dead *Daphnia* as described above. Infection status was recorded on day 23 (16 days after first exposure), day 39 and day 45 (18 days after the last exposure). Individuals dead before the first infection assessment were removed from the analyses; the status of those dead before the third infection assessment was considered to be the last recorded.

***Experiment 3: One or two exposures, three temperatures, two clones***

Hosts were exposed to each spore isolate either once (single exposure) or twice (double exposure), or to a control solution containing either deionised water or a macerated healthy daphnia, at 15ºC, 20ºC, or 25ºC. Prior to exposure, 30 replicate “maternal line” jars (5 *Daphnia* per jar) were maintained for three generations in a climate control chamber at 20ºC, 12:12 hour light:dark cycle, fed equal amounts of chemostat grown *Chlorella* sp. microalgae, and were changed into clean medium regularly to equilibrate any maternal effects on the hosts used in the infection. For the infection, the second clutch from third-generation of each maternal line replicate jar was split into the different treatments. Each infection replicate (Sp1or control; single or double exposure; 15ºC, 20ºC or 25ºC), received a single female (< 24 hours old) placed in a jar containing 60 ml of artificial *Daphnia* medium and a teaspoon of sterile sand. All infection jars were prepared in advance and kept in climate control chambers at the desired temperature overnight. There were 30 replicates per treatment, except for control treatments, which had 24 replicates per temperature, giving a total of 432 experimental jars. All infection replicates received 2500 *P. ramosa* spores on the first day and double exposure treatments received an additional 2500 spores on degree-day 120. The spore solutions were obtained by homogenising infected *Daphnia* in deionised water. Hence, control replicates received the equivalent volume of either a solution of crushed healthy *Daphnia* or deionised water (12 replicates each, per temperature). The infection period lasted 200-degree days, during which time all replicates (including controls) were stirred daily and fed low amounts of food (2.1 x10^6^ algae).

After the infection period, all replicate *Daphnia* were transferred to jars containing 60 ml artificial *Daphnia* medium, food levels were increased and they were maintained at the desired temperature (15ºC, 20ºC, or 25ºC) in climate control chambers (12:12 light:dark cycle). The number of offspring from each cutch was recorded until degree-day 700, and the medium changed with every clutch (or every 3 days if host reproduction ceased due to infection). Infection was assessed between degree-day 400-500. By this time infected individuals were red in colour, larger and had ceased reproducing. Hosts that died were collected, dried, and individually frozen at -20ºC in Eppendorf tubes. Host mortality was recorded until degree-day 1200, and the day of death was used to assess differences in survival between treatments. During the experiment, all replicate jars were randomly assigned to trays (24 jars per tray) and the position of jars within trays and of trays within incubators was changed regularly to equilibrate possible positional effects.

**A2: Infection costs**

***Experiment 1: Single exposure, four clones***

For the age of first reproduction, the Inf:Expo interaction was close to significance (*F*= 2.75, *P*= 0.06). This is due essentially to the clone GG3: for this clone, the individuals infected with the *P. ramosa* strain Sp1 (*a*, Fig. 1A) reproduced significantly earlier than uninfected individuals (*b*, Fig. 1A) or the controls (~2 days earlier, Sp1 Inf: *F*= 15.05, *P*< 0.001). No infection effect was found for the other genotypes on this trait (*F*= 1.37, *P*= 0.25). No effect of infection was found on the size at day 12 (*F*= 3.64, *P*= 0.06, Fig. 1B). Infected individuals lived significantly less long than uninfected (*F*= 4.99, *P*< 0.05), almost by half, but there was no effect of the type of exposure or of the genotype (*F*= 1.36, *P*= 0.24, Fig. 1C). Their fecundity was very low, with only 6 offspring on average (compare to an average of more than 80 for the uninfected). There was a small effect of the host-parasite combination, GG13 infected individuals managing to produce significantly more offspring (12 on average, *F*= 4.45, *P*= 0.037; they managed to produce a few more offspring after the establishment of the infection, P. Labbé, pers. obs.). Finally, the number of *P. ramosa* spores counted at the death of infected individuals was not different between neither exposure nor genotype, and was positively correlated to the age at death (as in Ebert et al. 2004, data not shown).

***Experiment 2: Multiple exposures, two clones***

After the first exposure, as expected, the infection level was not different between treatments, (14, 7 and 13 infected individuals for those exposed once, twice and four-times, respectively). However, no more infected individuals were detected in the twice exposure treatment, whereas 3 and 6 individuals were found after the third and the fourth exposure (resp.) in the four-time exposure treatment, indicating that some of the individuals which escaped the first exposures succumb to the later.

For the age of first reproduction (Fig. 2A) we found no effect of the infection status (Inf, *F*= 2.31, *P*= 0.13). The infected individuals died much earlier than the non-infected (Fig. 2B), with a significant trend for those exposed four times to die later (Expo, *F*= 4.70, *P*= 0.036), which is explain by the fact that those infected later (i.e. not on the first exposure) survive longer (data not shown). The infected individuals had a low fecundity (Fig. 2C, but higher than in the first experiment, probably due to the lower dose). Those exposed four times reproduced significantly more (Expo, *F*= 7.25, *P*= 0.002), again due to those getting infected only in the last exposures which reproduced for a longer period before being castrated. The analysis of other fecundity related traits (data not shown) confirmed these patterns. Finally, the number of *P. ramosa* spores counted at the death of infected individuals was significantly higher (*F*= 6.01, *P*= 0.018) for individuals exposed four times than for those exposed once or twice (Fig. A4).

***Experiment 3: One or two exposures, three temperatures, one clone***

We found a significant effect of temperature on the proportion of hosts that became infected (*χ^2^*=7.49, *P*= 0.024, Fig. A5). Double exposures resulted in higher infectivity at 20ºC and 25ºC (*χ^2^*= 4.36, *P*= 0.037), but not at 15ºC. Despite this, we did not find any significant effect of the dose of exposure (*χ^2^*= 0.39, *P*= 0.535) or a dose-by-temperature interaction (*χ^2^*= 4.55, *P*= 0.103).

**Bibliography**

Carius, H. J., T. J. Little, and D. Ebert. 2001. Genetic variation in a host-parasite association: potential for coevolution and frequency-dependent selection. Evolution 55:1136-1145.

Ebert, D., H. Joachim Carius, T. Little, and E. Decaestecker. 2004. The evolution of virulence when parasites cause host castration and gigantism. The American Naturalist 164:S19-S32.

**Table A3:** Infection levels for the different host-parasite combinations. A) For each pair of host clone and parasite strain, the expected percentage of infected hosts is given (modified from Carius et al. 2001). The “Av. inf.” column indicates for each *P. ramosa* strain the average percentage of infected hosts over 9 host clones, thus representing the infectivity level of the strain (a higher percentage indicates a higher infectivity). The “Av. res.” row indicates for each *D. magna* clone the average percentage of infected hosts over 9 parasite strains, thus representing the resistance level of the clone (a higher percentage indicates a lower resistance). B) This table present the percentage of infected hosts observed in Experiment 1 for the various combinations of host clones and parasite strains (for Sp 1, L and H indicates low and high dose, respectively).

A)

| **Parasite strain** | **Host clones** | | | |  |  |
| --- | --- | --- | --- | --- | --- | --- |
|  | **3** | **4** | **7** | **13** |  | **Av. inf.** |
| **1** | 60-80 | 80-100 | 0 | 0-20 |  | 46 |
| **8** | 60-80 | 0 | 0 | 20-40 |  | 24 |
| **Av. res.** | 91 | 73 | 28 | 17 |  |  |

B)

| **Parasite strain** | **Host clones** | | | | |
| --- | --- | --- | --- | --- | --- |
|  | **3** | **4** | **7** | **13** |  |
| **1** | L: 74.3  H: 68.6 | L: 61.8  H: 79.4 | L: 0  H: 0 | L: 0  H: 0 |  |
| **8** | 57.1 | 0 | 0 | 60.0 |  |

**Fig A4:** Experiment 2 (two genotypes, multiple exposures) spore loads. Here is presented the number of spores counted in infected GG4 hosts on the day of death; the order from lighter to darker grey is individuals exposed once, twice or four-times. Errors bars represent the standard error.

**Fig A5:** Experiment 3 (one or two exposures, three temperatures, one clone) spore loads. Here is presented the proportion of infected individuals at different temperatures; white and grey bars represent the individuals exposed to a single or a double dose, respectively. Errors bars represent the standard error.
